# Hypertension crisis as the first symptom of renovascular hypertension in children

**DOI:** 10.1186/s13052-022-01378-4

**Published:** 2022-12-02

**Authors:** Lingling Xu, Hongjun Ba, Xiaoyun Jiang, Youzhen Qin

**Affiliations:** 1 Department of Pediatrics, The First Affiliated Hospital of Sun Yat-Sen University, 58 Zhongshan Second Road, Guangzhou, Guangdong 510080 People’s Republic of China; 2 Department of Pediatric Cardiovascular, The First Affiliated Hospital of Sun Yat-Sen University, 58 Zhongshan Second Road, Guangzhou, Guangdong 510080 People’s Republic of China

**Keywords:** Renovascular hypertension, Children, Hypertensive crisis, Hypertensive urgency, Hypertensive emergency

## Abstract

**Background:**

Renovascular hypertension (RVH) is one of the main causes of hypertensive crisis (HTN-C). It is characterized by acute onset and severe disease, and early diagnosis and treatment are difficult. The objective was to describe the characteristics of RVH and factors associated with RVH leading to HTN-C in children. At present, there are few clinical studies on RVH in children with large cases in China.

**Methods:**

This study retrospectively analyzed the clinical data of inpatient children with RVH. Patients were divided into non-hypertensive crisis (non-HTN-C) group, and HTN-C group according to the first symptoms and blood pressure. Further, HTN-C were classified as hypertensive urgency (HTN-U) or hypertensive emergency (HTN-E).

**Results:**

Fifty-four pediatric cases (41 boys and 13 girls) were included. 83.3% of the RVH cases were ≥ 6 years old. Three cases were classified into the non-HTN-C group. Of the 51 HTN-C cases, 18 cases were grouped as HTN-U and 33 as HTN-E. The HTN-U group were mainly asymptomatic (50.0%, 9/18) while the HTN-E group mainly presented with neurological symptoms (72.7%, 24/33). The number of unknown etiology children was 32 (59.2%). The top three known etiologies were Takayasu’s arteritis (50.0%, 11/22), congenital renal dysplasia (27.3%, 6/22) and fibromuscular dysplasia (13.6%, 3/22). As for the target organ damage of RVH, patients had a higher prevalence of left ventricular hypertrophy (71.4%, 35/49) and retinopathy (77.8%, 21/27).

**Conclusions:**

Most RVH patients with HTN-C as the first symptoms, especially for males over 6 years old, should be assessed for RVH even if they were asymptomatic. Most asymptomatic patients with RVH already had target organ damage, and symptomatic patients even developed life-threatening complications. As preventive measures, routine monitoring of BP during children’s physical examinations is advised.

## Introduction

Hypertension is a global health hazard. Hypertension present a higher prevalence and patients tend to be younger. In recent years, the incidence of hypertension in children has been increasing. Severe hypertension presents a potentially life-threatening condition. One of the most commonly used term to define severe hypertension is hypertensive crisis (HTN-C), a sudden and severe increase in blood pressure above baseline levels, leading to rapid end-organs damage which could be life-threatening. HTN-C is rare in children but if it is not timely diagnosed or left untreated, irreversible damage to vital organs could occur. At present, there is no unified definition of HTN-C in children and adolescents. Several HTN-C definitions had been used in literature, including “blood pressure well above the 99th percentile”[1], or “stage 2 hypertension”[2], or “20 mmHg above the 95th percentile”[3], or “a cutoff of 20% above the stage 2 hypertension limit”[4]. Further, HTN-C has also been classified as hypertensive urgency (HTN-U) or hypertensive emergency (HTN-E), depending on end-organ involvement including cardiac, renal and neurologic injury. There are many causes of HTN-C whereby renovascular hypertension (RVH) in secondary hypertension is one of the main causes [5, 6].

RVH is the most important cause of secondary hypertension in children resulting from renal artery stenosis (RAS) or occlusion of the main renal artery and/or its branches. RVH accounts for about 5%-10% of the total cases of hypertension in children [7, 8]. It has a rapid onset, mostly manifesting as HTN-C and/or refractory hypertension, and can lead to hypertensive target organ damage. Thus, early diagnosis and timely active treatment of RVH is very important as delays could cause irreversible damage to vital organs such as the heart, brain, kidneys and eyes.

Currently, compared with adults’ RVH. There are few clinical studies on RVH in children with large cases in China. Further, considering the lack of specificity of RVH symptoms and that not routinely measuring the blood pressure of children during routine treatment are important factors delaying timely diagnosis and treatment of RVH, this study intends to review retrospectively the children with renovascular hypertension diagnosed in our hospital, collect clinical data and analyze their clinical features in order to summarize their clinical manifestations and target organ damage, to provide theoretical basis for the diagnosis and treatment of renovascular hypertension in children in the future.

## Materials and methods

### Methods of retrospective case review

The electronic case query system of the First Affiliated Hospital of Sun Yat-Sen University (Guangzhou, China) was used to retrieve cases, aged ≤ 18 years old, diagnosed with RVH from January 1991 to February 2019. Important data required for inclusion were: (a) Age of onset (≤ 18 years old) and gender; (b) Details of the first onset of hypertension symptoms such as headache, dizziness, blurred vision, palpitation, oliguria, hemiplegia and convulsion; (c) Time from the first symptom to diagnosis and past disease history before confirmed diagnosis; (d) Blood pressure level at first admission; (e) Presence of renal vascular stenosis and imaging examination results before invasive treatment; (f) Plasma renin, angiotensin, aldosterone and blood potassium level; (g) Diagnosis and treatment information.

Repeated admissions were excluded.Renal artery graft stenosis causing hypertension were excluded.

### Diagnostic criteria for RVH in children

All cases should have: 1) met the diagnostic criteria for hypertension [1]: (a) for children aged 1–13 years old, hypertension was defined as average clinic measurement of SBP and/or DBP ≥ 95th percentile (based on age, sex, and height percentiles); (b) for children aged ≥ 13 years old, hypertension was defined as average clinic measured BP ≥ 130/80 mmHg; 2) had imaging examination showing the degree of stenosis (≥ 50%) of the main or main branches of the renal artery [9].

### Diagnostic criteria for HTN-C in children

HTN-C was defined as cases with persistent BP > 99th percentile (based on age, sex, and height percentiles) [1], or severe hypertension of any age with BP > 150/100 mmHg with or without acute end-organ failure or dysfunction. HTN-E was defined as cases with symptoms of acute end-organ failure or dysfunction. Cases without acute end target organ failure or dysfunction were defined as HTN-U.

### Diagnostic criteria for Takayasu’s arteritis (TA)

Diagnosis of TA was based on the European League against Rheumatism and Paediatric Rheumatology European Society (EULAR/PReS) endorsed consensus criteria for the classification of childhood vasculitides [10].

### Diagnostic criteria for fibromuscular dysplasia (FMD)

Renal artery FMD was defined as the presence of the typical string of beads appearance, or the presence of unique stenosis of the renal artery [11].

### Assessment of target organ damage

Cerebral and ocular complications of RVH were assessed via head imaging and fundus examination. Left ventricular hypertrophy (LVH) was defined as a left ventricular mass index greater than the 95th percentile as per age-specific centiles.

### Patients’ grouping

According to the blood pressure level (such as BP > 150/100 mmHg or persistent BP > 99th) and clinical manifestations (such as convulsion, blurred vision) at the first visit, the cases were classified into a non-hypertensive crisis (non-HTN-C), HTN-U and HTN-E group. The differences in clinical characteristics and diagnostic examinations of each group were assessed.

### Response to intervention

The therapy results of RVH after the last treatment were categorized as cured (blood pressure reduced to below the 95th percentile without requiring antihypertensive medications); improved (blood pressure reduced below 95th percentile but still required antihypertensive medications or DBP reduced by more than 15% of pre-intervention level), and; failed (hypertension persisted despite antihypertensive medications with less than a 15% decrease in DBP from pre-intervention level). Blood pressure at the most recent follow-up was obtained from clinical outpatient records.

### Statistical analysis

Continuous measurement data are expressed as means ± standard deviation. Enumeration data are expressed as a percentage of the total counts. The data distribution of each covariate among each group was compared using the Kruskal–Wallis rank-sum test (non-normal distribution) for continuous variables and Fisher's exact probability for enumeration data (Counting variables having theoretical numbers < 10). *P* values < 0.05 were considered as having statistical significance. All analyses were performed using the R (www.R-project.org) and EmpowerStats (www.empowerstats.com, X&Y solutions, Inc. Boston MA) software.

## Results

### Study participants

In all, 54 cases of children (41 males and 13 females) diagnosed with RVH during their hospitalization at the First Affiliated Hospital of Sun Yat-sen University from January 1991 to February 2019 were eligible for this study.

### Demographics of RVH children with or without HTN-C

RVH not caused by transplant renal artery stenosis in children accounted for 6.8% (54/792) of secondary hypertension. The clinical and laboratory characteristics of the included RVH patients are presented in Table 1. Among them, males were more prone to hypertensive crises. The ratio of males to females was nearly 3:1. Based on the severity of hypertension of the 54 included cases, they were first categorized into an HTN-C (51/54 cases; 94.4%) and non-HTN-C group (3/54 cases; 5.6%). Among the HTN-C cases, the detection rate of HTN-C was 16.7% before the age of 6, 83.3% after the age of 6, and 46.3% after the age of 12. Next, the HTN-C group was further categorized into an HTN-U (18/51 cases; 35.3%) and HTN-E (33/51 cases; 64.7%) group. Significant differences (*P* = 0.006) in SBP levels, DBP levels and the first onset of symptoms of hypertension among the three groups (non-HTN-C, HTN-U and HTN-E) were observed (Table 1).

**Table 1:** Clinical characteristics of 54 children with RVH

Variables	Total	Non-HTN‐C	HTN‐C		*P*-value
			**HTN‐U**	**HTN‐E**	
No. of cases	**54**	3	18	33	
Sex (n,%)					**0.916**
Female	**13 (24.1)**	1 (33.3)	4 (22.2)	8 (24.2)	
Male	**41 (75.9)**	2 (66.7)	14 (77.8)	25 (75.8)	
BP (mmHg)					
SBP	**180.2 ± 31.2**	140.3 ± 19.5	169.9 ± 18.3	189.5 ± 33.4	**0.006**
DBP	**113.0 ± 25.2**	85.7 ± 6.7	107.6 ± 21.4	118.4 ± 26.3	**0.051**
Different age groups at admission (years) (n,%)					**0.429**
< 6	**9 (16.7)**	1 (33.3)	4 (22.2)	4 (12.1)	
6–12	**20 (37.0)**	0 (0.0)	5 (27.8)	15 (45.5)	
12–18	**25 (46.3)**	2 (66.7)	9 (50.0)	14 (42.4)	
Age of admission (year)	**11.3 ± 4.7**	15.0 ± 3.3	11.0 ± 5.2	11.2 ± 4.4	**0.387**
Age of first symptoms (year)	**10.6 ± 4.7**	14.9 ± 3.3	10.4 ± 5.1	10.4 ± 4.5	**0.272**
Age of diagnosis RVH (year)	**11.3 ± 4.5**	15.0 ± 3.3	11.0 ± 5.3	11.2 ± 4.1	**0.364**
TT admission RVH (month)	**2.7 (0.00–120.0)**	1.2 (0.1–2.0)	2.0 (0.2–52.6)	4.0 (0.0–120.0)	**0.530**
TT symptoms admission (month)	**3.1 (0.0–53.1)**	0.9 (0.0–2.1)	2.2 (0.0–53.1)	4.9 (0.1–53.1)	**0.5545**
First symptoms (n,%)					**0.001**
Acute kidney injury	**3 (5.6)**	0 (0.0)	3 (16.7)	0 (0.0)	
Asymptomatic	**14 (25.9)**	2 (66.7)	9 (50.0)	3 (9.1)	
Heart failure	**7 (13.0)**	0 (0.0)	1 (5.6)	6 (18.2)	
Neurological symptoms	**30 (55.6)**	1 (33.3)	5 (27.8)	24 (72.7)	
Headache	**21(38.9)**	*1(25.0)*	*4(22.2)*	*16(48.5)*	
Blurred vision	**10(18.5)**	*0(0.0)*	*0(0.0)*	*10(30.3)*	
Dizzy	**7 (12.9)**	*1(25.0)*	*1(5.0)*	*5(15.2)*	
Convulsion	**4 (7.4)**	*0(0.0)*	*0(0.0)*	*4(12.1)*	
Recurrent syncope	**1 (1.9)**	*0(0.0)*	*0(0.0)*	*1(3.0)*	
Hemiplegia	**1 (1.9)**	*0(0.0)*	*0(0.0)*	*1(3.0)*	
Limb weakness	**1 (1.9)**	*0(0.0)*	*0(0.0)*	*1(3.0)*	
Etiology (n,%)					**0.666**
Atherosclerosis	**1 (4.5)**	0 (0.0)	0 (0.0)	1 (7.7)	
Congenital renal dysplasia	**6 (27.3)**	0 (0.0)	2 (28.6)	4 (30.8)	
FMD	**3 (13.6)**	1 (50.0)	1 (14.3)	1 (7.7)	
TA	**11 (50.0)**	1 (50.0)	3 (42.9)	7 (53.8)	
Trauma	**1 (4.5)**	0 (0.0)	1 (14.3)	0 (0.0)	
Other vascular abnormalities (n,%)					**0.602**
No	**46 (85.2)**	2 (66.7)	16 (88.9)	28 (84.8)	
Yes	**8 (14.8)**	1 (33.3)	2 (11.1)	5 (15.2)	
Stenosis side (n,%)					**0.181**
Bilateral	**12 (22.2)**	1 (33.3)	2 (11.1)	9 (27.3)	
Left	**21 (38.9)**	0 (0.0)	6 (33.3)	15 (45.5)	
Right	**21 (38.9)**	2 (66.7)	10 (55.6)	9 (27.3)	
Site of stenosis (n,%)					**0.686**
Proximal	**19 (59.4)**	3 (100.0)	5 (71.4)	11 (50.0)	
Distal	**4 (12.5)**	0 (0.0)	1 (14.3)	3 (13.6)	
middle	**6 (18.8)**	0 (0.0)	1 (14.3)	5 (22.7)	
Full	**3 (9.4)**	0 (0.0)	0 (0.0)	3 (13.6)	
Inequality of renal size (> 1.5 cm difference) (n,%)					**0.457**
No	**42 (82.4)**	3 (100.0)	15 (88.2)	24 (77.4)	
Yes	**9 (17.6)**	0 (0.0)	2 (11.8)	7 (22.6)	
High plasma renin and Ang II levels (n,%)					**0.596**
No	**8 (21.6)**	1 (50.0)	2 (18.2)	5 (20.8)	
Yes	**29 (78.4)**	1 (50.0)	9 (81.8)	19 (79.2)	
Hyponatremia (n,%)					**0.827**
No	**49 (90.7)**	3 (100.0)	16 (88.9)	30 (90.9)	
Yes	**5 (9.3)**	0 (0.0)	2 (11.1)	3 (9.1)	
Hypokalemia (n,%)					**0.581**
No	**41 (75.9)**	3 (100.0)	13 (72.2)	25 (75.8)	
Yes	**13 (24.1)**	0 (0.0)	5 (27.8)	8 (24.2)	

*Abbreviations*: *RVH* Renovascular hypertension, *Non-HTN-C* Nonhypertensive crisis, *HTN‐C* Hypertensive crisis, *HTN‐U* Hypertensive urgency, *HTN-E* Hypertensive emergency, *N* Number of cases, *BP* Blood pressure, *SBP* Systolic blood pressure, *DBP* Diastolic blood pressure, *y* year/years, *TT Admission RVH* Time from admission to RVH diagnosis, *TT symptoms admission* Time from symptoms to admission, *FMD* Fibromuscular dysplasia, *TA* Takayasu's arteritis, *Ang II* Angiotensin II

### Distribution trends of RVH across different years and months

**Fig. 1:**
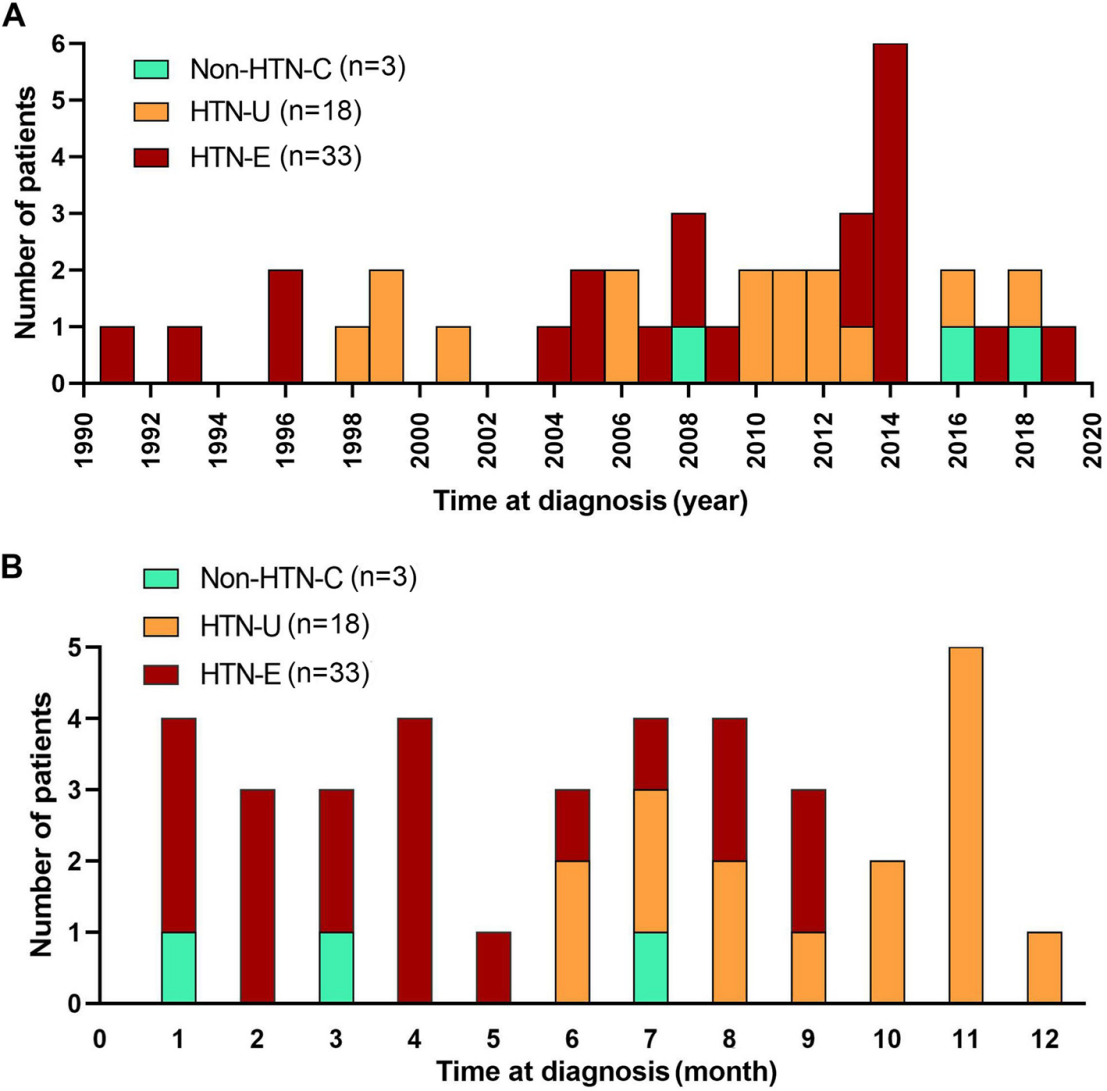
Distribution of hypertension in RVH children by year, from 1991 to 2019 (**A**), and by months (**B**)

With the development of medical technology, more and more RVH cases were diagnosed. The number of children diagnosed with RVH varied from month to month, but a trend of fewer cases of RVH diagnosed in May and December each year were also observed (Fig. 1).

### Clinical manifestations of RVH in children

**Fig. 2:**
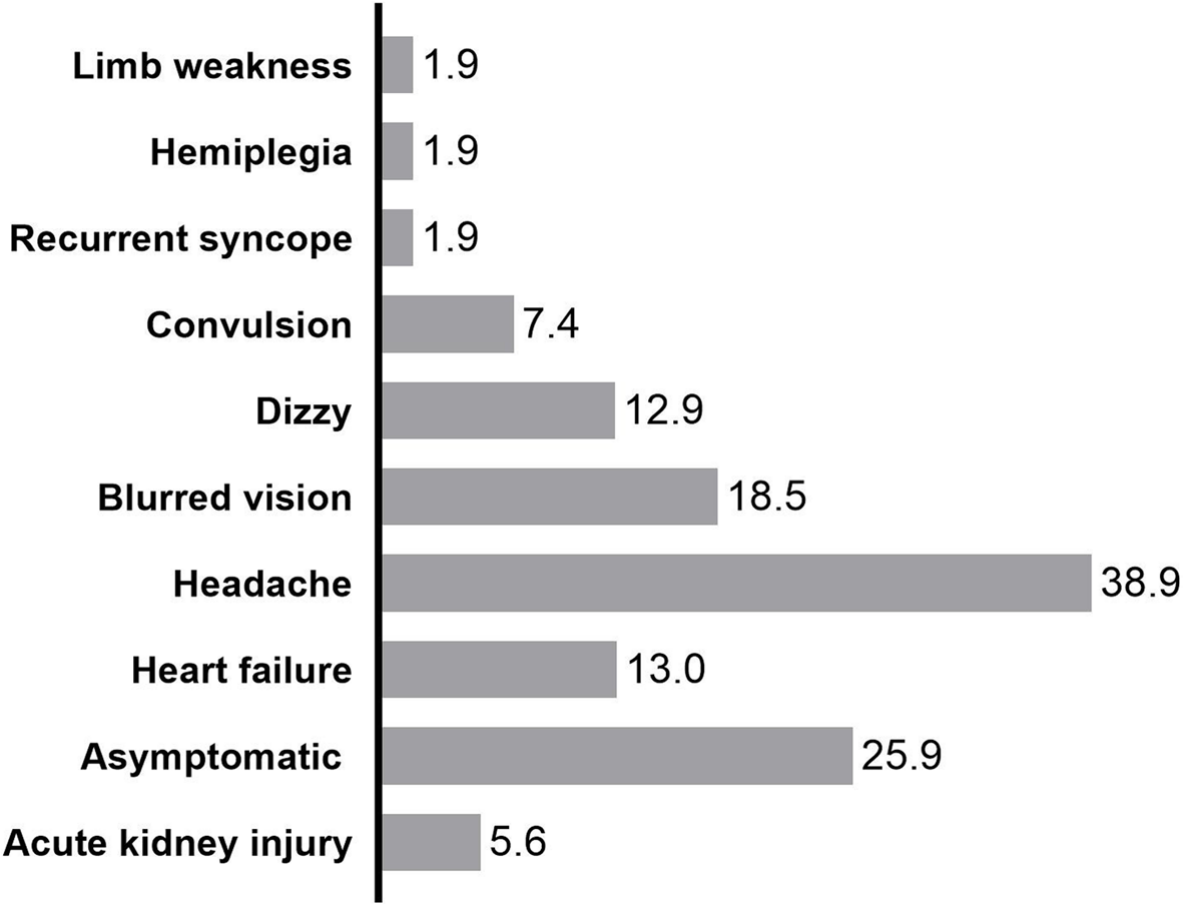
Ratio of clinical manifestations at the time of RVH diagnosis in pediatric patients

Among the 54 children with RVH, 25.9% (14/54) were asymptomatic and 55.6% (30/54) presented with neurological symptoms, including headache (21/54 cases; 38.9%), blurred vision (10/54 cases; 18.5%), dizziness (7/54 cases; 13.0%), and convulsion (4/54 cases; 7.4%) (Fig. 2). Fourteen patients were asymptomatic, among which 7 patients were found to have high blood pressure by physical examination, and 7 patients were found to have high blood pressure by blood pressure measurement when they needed hospital treatment due to adenoid hyperplasia, scoliosis, respiratory tract infection, etc.

When children had neurological symptoms, 16.7% (5/30) presented with HTN-U and 80.0% (24/30) with HTN-E. The first symptoms of children with hypertensive emergencies were headache in 48.5% (16/33) of cases, blurred vision in 30.3% (10/33) of cases, dizziness in 15.2% (5/33) of cases, heart failure in 18.2% (6/33) of cases, and convulsion in 12.1% (4/33) of cases.

### Etiology of RAS in children

**Fig. 3:**
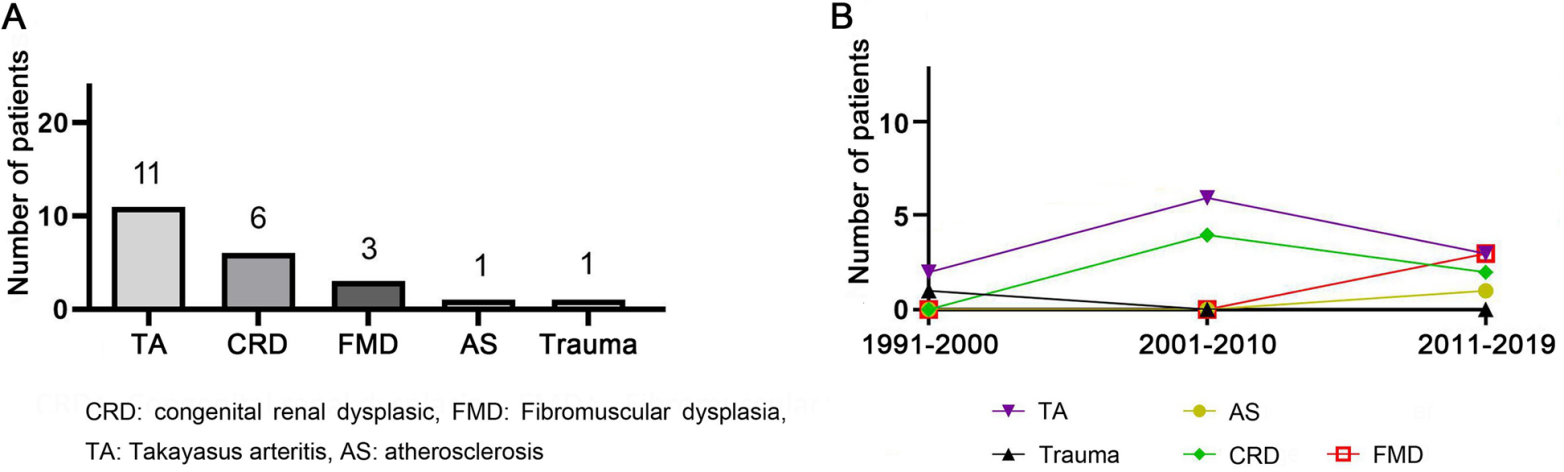
Incidence of RVH based on etiology (**A**) and at three different time periods (**B**)

The etiology of 22 cases was confirmed. Among the 22 RVH cases, 11 cases were due to Takayasu arteritis, 6 due to congenital renal dysplasia, 3 due to FMD, and 1 due to atherosclerosis. There was also one case of RAS after a car accident (Fig. 3).

### Target organ damage and treatment of RVH in children

**Fig. 4:**
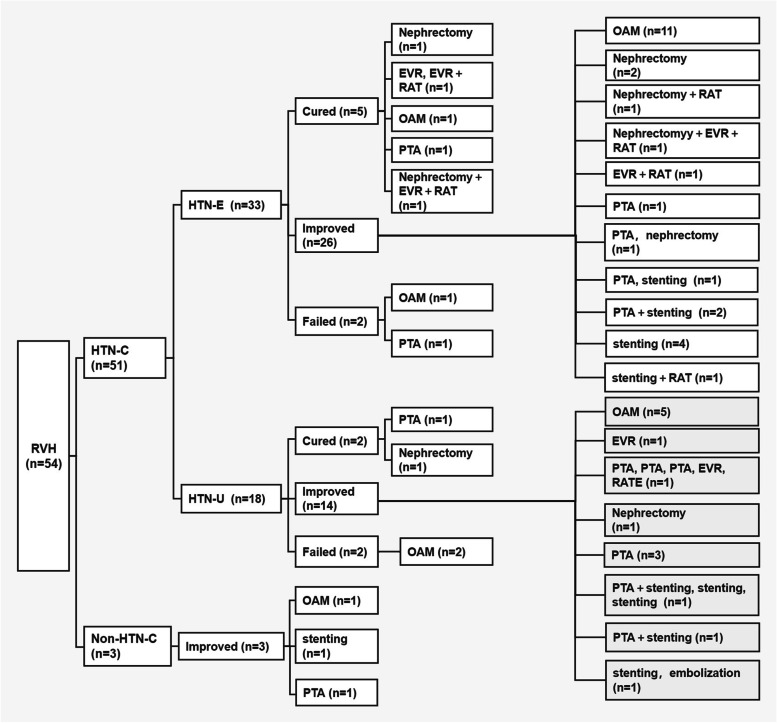
The treatment and outcomes of RVH cases in groups. Note: Classification based on diagnosis: RVH was classified as HTN-C and non-HTN-C. HTN-C was further classified as HTN-E and HTN-U. Classification based on treatment outcomes: Cured, BP reduced to below the 95th percentile without requiring antihypertensive medications; Improved, BP reduced to below 95th percentile but still required antihypertensive medications or DBP reduced by more than 15% of pre-intervention level; Failed, hypertension persisted despite antihypertensive medications with less than a 15% decrease in DBP from pre-intervention level. Abbreviations: RVH: renovascular hypertension; Non-HTN-C: Nonhypertensive crisis; HTN‐C: hypertensive crisis; HTN‐U: hypertensive urgency; HTN-E: hypertensive emergency; OAM: Only antihypertensive medication; PTA: Percutaneous transluminal angioplasty; RAT: renal autotransplantation; EVR: endovascular repair; RATE: Renal artery thrombectomy

Data regarding target organ damage and corresponding treatments of the 54 children with RVH are presented in Table 2. In addition, 9.3% (5/54) of the children with RVH had cerebral hemorrhage, 3.7% (2/54) had retinal hemorrhage, 71.4% (35/49) had left ventricular hypertrophy, 40.0% (20/50) had hypertensive encephalopathy, 77.8% (21/27) had retinopathy, 18.5% (10/54) had abnormal renal function, and 37.0% (20/54) had proteinuria. In regards to treatments, 38.9% (21/54) had only medication for antihypertensive therapy, 38.9% (21/54) underwent angioplasty, and 22.2% (12/54) underwent surgical treatment. After treatment, 7 cases were cured, 43 cases had improved symptomatic treatments (such as nephrectomy, renal autotransplantation, endovascular repair and renal artery thrombectomy), and 4 were non-responsive to treatments (Fig. 4). Five cases were admitted with cerebral hemorrhage, and were treated with intravenous antihypertensive followed by symptomatic management and angioplasty after their condition stabilized.

**Table 2:** Target organ damage and treatment of 54 children with RVH

Variables	Total	Non-HTN‐C	HTN‐C		*P*-value
			HTN‐U	HTN‐E	
Cerebral hemorrhage (n,%)					0.173
No	49 (90.7)	3 (100.0)	18 (100.0)	28 (84.8)	
Yes	5 (9.3)	0 (0.0)	0 (0.0)	5 (15.2)	
Retinal hemorrhage (n,%)					0.516
No	52 (96.3)	3 (100.0)	18 (100.0)	31 (93.9)	
Yes	2 (3.7)	0 (0.0)	0 (0.0)	2 (6.1)	
Left ventricular hypertrophy (n,%)					0.024
No	14 (28.6)	2 (100.0)	6 (40.0)	6 (18.8)	
Yes	35 (71.4)	0 (0.0)	9 (60.0)	26 (81.2)	
Hypertensive encephalopathy (n,%)					< 0.001
No	30 (60.0)	2 (100.0)	16 (100.0)	12 (37.5)	
Yes	20 (40.0)	0 (0.0)	0 (0.0)	20 (62.5)	
Retinopathy (n,%)					< 0.001
No	6 (22.2)	1 (100.0)	4 (100.0)	1 (4.5)	
Yes	21 (77.8)	0 (0.0)	0 (0.0)	21 (95.5)	
Abnormal renal function (n,%)					0.654
No	44 (81.5)	3 (100.0)	14 (77.8)	27 (81.8)	
Yes	10 (18.5)	0 (0.0)	4 (22.2)	6 (18.2)	
Proteinuria (n,%)					0.975
No	34 (63.0)	2 (66.7)	11 (61.1)	21 (63.6)	
Yes	20 (37.0)	1 (33.3)	7 (38.9)	12 (36.4)	
Treatment modality (n,%)					0.677
Only medication	21 (38.9)	1 (33.3)	7 (38.9)	13 (39.4)	
Angioplasty	21 (38.9)	2 (66.7)	8 (44.4)	11 (33.3)	
Surgery	12 (22.2)	0 (0.0)	3 (16.7)	9 (27.3)	
Outcome (n,%)					0.851
Cured	7 (13.0)	0 (0.0)	2 (11.1)	5 (15.2)	
Improved	43 (79.6)	3 (100.0)	14 (77.8)	26 (78.8)	
Failed	4 (7.4)	0 (0.0)	2 (11.1)	2 (6.1)	

*Abbreviations*: *RVH* Renovascular hypertension, *Non-HTN-C* Nonhypertensive crisis, *HTN‐C* Hypertensive crisis, *HTN‐U* Hypertensive urgency, *HTN-E* Hypertensive emergency, *LVH* Left ventricular hypertrophy

## Discussion

HTN-C is a relatively rare event and is associated with end-organ damage. One study has reported that 18.9% of children with HTN-C were caused by RAS [12]. The incidence of RVH in both adults and pediatrics is about 5%-10% [8, 13]. The difference between RVH and other causes of hypertension is that RVH can be cured by medication, intravascular intervention or surgery. In this study, we evaluated the clinical manifestations, diagnosis, treatment, and therapy results of 54 patients with heterogeneous renal artery disease leading to pediatric RVH, and represents one of the largest series of pediatric RAS studies to date, besides studies from Lobeck et al. [14], Agrawal et al. [15] and Wu et al. [16].

This study found that the detection rate of RVH in children was 5.5%, accounting for 6.8% (54/792) of 792 children with secondary hypertension. 83.3% of the RVH patients were male children over 6 years old. The ratio of male to female was about 3:1, which is consistent with a previous study[8]. We observed that the detection rate of HTN-C in children with RVH was as high as 94.4%, of whom 64.7% (33/51) were hypertensive emergencies. Lee et al. [5] reported RVH as the main cause of HTN-C, indicating that despite the incidence of renovascular hypertension may not be high, however, its clinical manifestations could be life-threatening if not diagnosed and treated on time. For children with hypertension, proper awareness and guidance should be provided to the caretaker regarding the first manifestation of HTN-C and male children for a timely diagnosis of RVH.

The clinical manifestations of RVH in children may vary greatly. They may be asymptomatic, or may have obvious headaches, dizziness, and even severe neurological symptoms such as convulsions and hemiplegia. Similar to the results reported by Lobeck et al.[14], the vast majority of children with RVH in this study had HTN-C at the time of treatment, and the main symptoms were hypertensive emergencies. We also found that most of the children in the HTN-U group were asymptomatic. Therefore, we suggest that it is necessary to monitor the blood pressure of children during routine physical examinations to screen for hypertension. The first symptoms of the HTN-E group were headache and blurred vision as the main neurological symptoms, which is consistent with a previous report [14]. Moreover, the HTN-E group had higher SBP and DBP than the HTN-U group, and the SBP was higher and more obvious than the HTN-U group. The difference between HTN-E and HTN-U groups was statistically significant, which is consistent with the results of a study by the Institute of Clinical Medicine in Taiwan [16]. Therefore, for severe hypertension with neurological symptoms, it is necessary to provide timely blood pressure control, especially for elevated SBP, and actively screen for RVH to avoid progression to hypertensive emergency.

Our study found that 77.8% (21/27) of children with RVH had retinopathy, 40.0% had hypertensive encephalopathy and 71.4% had left ventricular hypertrophy, which was consistent with the results reported by Tullus et al. [8]. These were higher than the detection rates of retinopathy [17], hypertensive encephalopathy [16], and left ventricular hypertrophy reported in previous literatures and could be related to ethnic differences and the high salt diet habits of Chinese children [5, 18]. Further, because most of the children with RVH in this study were asymptomatic and were found to have high blood pressure during outpatient clinics or physical examinations (or even reached sub-emergency hypertension), lack of awareness among their parents could have contributed to delayed treatments, for several months or even more than a year, before a formal diagnosis and treatment of hypertension. Some patients after having obvious headaches and other neurological symptoms were not properly treated, leading to the majority of children with RVH in this study who were in the HTN-C group, and target organ damage being more common in them. Therefore, children with RVH should be routinely evaluated for target organ damage.

RAS is mostly caused by conditions such as FMD, Takayasu’s arteritis, neurofibromatosis as well as some unexplained factors that lead to RAS. Tullus et al. [8] reported that there could be variable etiologies of RVH in children, and could also be associated with regional differences. FMD is the most common cause of RVH in children in North America and Europe, with an incidence rate of 35%-76% [14, 19, 20]. However, Takayasu's arteritis is the main cause of RVH in children in Asia and South Africa [8]. McCulloch et al. [21] reported that 89% of children with RVH were mainly caused by TA. Studies on children with RVH in China and Turkey reported that the incidence of Takayasu's arteritis was 60%-72% [22, 23]. In this current study, we found that 59.3% (32/54) of children with RVH were of unknown etiology. Among the identified causes, Takayasu’s arteritis was the main cause, accounting for 50%, followed by congenital renal dysplasia (27.3%) then FMD (13.6%), and was different from the incidence of the etiology reported in a previous study[8]; and could indeed be related to the unknown etiology and regional differences of most patients in this study.

The arterial lesions of RVH can be different. About half of patients with RVH have bilateral renal artery involvement, with or without intrarenal and (or) extrarenal vascular disease [8]. In this study, the incidence of bilateral RAS was 22.2%, which was similar to the incidence of 24%-28% reported in other literatures [14, 24]. It was also observed in our study that 14.8% (8/54) of children with RVH had extracranial and intracranial cerebrovascular diseases, and most of the RAS was mainly proximal stenosis.

RVH is considered to be the prototype of renin-dependent hypertension. Therefore, the measurement of basal and stimulating plasma renin activity (PRA) in peripheral and renal venous blood is widely used for diagnosis. However, the findings from a study [25] showed that the release of renin on the stenotic side of RAS required 10% to 20% of the aortic-renal pressure gradient. When the pressure gradient reached 50%, the renal vein blood renin release of the stenotic side was the largest. This study found that not all children had elevated blood renin levels, which was inconsistent with the severity of renal artery stenosis. Unilateral RAS was the main cause of RVH and it was mostly related to peripheral venous blood sampling to detect renin levels. This indicates that low levels of peripheral blood or renal venous blood renin levels could not rule out RVH. Hypokalemia and increased plasma renin and angiotensin levels are considered to be clues to the diagnosis of RVH [8]. In this study, 13 children with RVH had hypokalemia, and most of them were accompanied by high renin levels. This further confirmed that the diagnosis of renovascular hypertension should be carefully assessed, when hypertension is complicated with hypokalemia. Therefore, our study findings may provide clues to improve the diagnosis and treatment of pediatric RVH.

### Limitations

The main limitation of this study is its retrospective single-institution design, which involved only hospitalized patients, spanned on about three decades and included different practice models, possibly leading to some extent of selection biases. Further, as this was a retrospective study, laboratory results were incomplete in some cases, and none of the cases could be suggested to undergo genetic testing, leading to a possible failure to diagnose NF1 and Williams' diseases. Also, some cases could not be included due to limited availability of early part of their electronic records for patient retrieval and assessments using diagnostic codes.

## Conclusion

In this study, RVH in children was commonly observed in males aged > 6 years old. Renovascular hypertension in children was more common in boys over 6 years old. 94.4% children were presented with hypertensive crisis. Clinical manifestations were varied, 55.6% cases had neurological symptoms, 25.9% cases were asymptomatic.SBP and DBP were significantly higher in HTN-E cases than in HTN-U cases. Takayasu’s arteritis was the main cause for known etiology in our study. Target organ damage in children with RVH were common. Seventy one point four percent cases have left ventricular hypertrophy, 77.8% cases have retinopathy and 40% cases have hypertensive encephalopathy. These target organ damage were asymptomatic in the early stage. The children with RVH were mainly treated with only antihypertensive drugs (38.9%), angioplasty (38.9%) and surgery (22.2%). Seventy nine point six percent cases were improved and 13.0% were cured.

## Data Availability

All datasets generated for this study are included in the manuscript.

## Abbreviations

Ang II: Angiotensin II
BP: Blood pressure
DBP: Diastolic blood pressure
EVR: Endovascular repair
FMD: Fibromuscular dysplasia
HTN‐C: Hypertensive crisis
HTN-E: Hypertensive emergency
HTN‐U: Hypertensive urgency
RVH: Renovascular hypertension
LVH: Left ventricular hypertrophy
Non-HTN-C: Nonhypertensive crisis
OAM: Only antihypertensive medication
PTA: Percutaneous transluminal angioplasty
RAS: Renal artery stenosis
RAT: Renal autotransplantation
RATE: Renal artery thrombectomy
SBP: Systolic blood pressure
TA: Takayasu’s arteritis

## References

[1] Subcommittee on Screening and Management of High Blood Pressure in Children. Clinical Practice Guideline for Screening and Management of High Blood Pressure in Children and Adolescents. Pediatrics. 2017;140:e20171904. doi: 10.1542/peds.2017-1904. 10.1542/peds.2017-1904 28827377

[2] Flynn, Joseph T, and Kjell Tullus. Severe hypertension in children and adolescents: pathophysiology and treatment. Pediatr Nephrol. 2009;24(6):1101–12. doi: 10.1007/s00467-008-1000-1. Epub 2008 Oct 7. 10.1007/s00467-008-1000-1 18839219

[3] Chandar J, Zilleruelo G (2012). Hypertensive crisis in children. Pediatr Nephrol.

[4] Lurbe I Ferrer, Empar. 2016 - Guías europeas para el manejo de la hipertensión arterial en niños y adolescentes: nuevos conceptos para un viejo problema” [2016-European Society of Hypertension Guidelines for the management of high blood pressure in children and adolescents]. An Pediatr (Barc) . 2016;85(4):167–9. doi: 10.1016/j.anpedi.2016.08.001. 10.1016/j.anpedi.2016.08.001 27692099

[5] Lee Y, Lim YS, Lee ST, Cho H. Pediatric renovascular hypertension: Treatment outcome according to underlying disease. Pediatr Int. 2018;60(3):264-9. 10.1111/ped.13491. 10.1111/ped.13491 29281158

[6] Gupta-Malhotra M, Banker A, Shete S, Hashmi SS, Tyson JE, Barratt MS (2015). Essential hypertension vs. secondary hypertension among children. Am J Hypertens.

[7] Wyszyńska T, Cichocka E, Wieteska-Klimczak A, Jobs K, Januszewicz P (1992). A single pediatric center experience with 1025 children with hypertension. Acta Paediatr.

[8] Tullus K, Brennan E, Hamilton G, Lord R, McLaren CA, Marks SD (2008). Renovascular hypertension in children. Lancet.

[9] Martin LG, Rundback JH, Sacks D, Cardella JF, Rees CR (2002). Quality improvement guidelines for angiography, angioplasty, and stent placement in the diagnosis and treatment of renal artery stenosis in adults. J Vasc Interv Radiol.

[10] Ozen S, Ruperto N, Dillon MJ, Bagga A, Barron K, Davin JC, Kawasaki T, Lindsley C, Petty RE, Prieur AM, Ravelli A, Woo P (2006). EULAR/PReS endorsed consensus criteria for the classification of childhood vasculitides. Ann Rheum Dis.

[11] Pasquini M, Trystram D, Nokam G, Gobin-Metteil MP, Oppenheim C, Touzé E (2015). Fibromuscular dysplasia of cervicocephalic arteries: Prevalence of multisite involvement and prognosis. Rev Neurol (Paris).

[12] Lim AM, Chong SL, Ng YH, Chan YH, Lee JH (2020). Epidemiology and Management of Children with Hypertensive Crisis: A Single-Center Experience. J Pediatr Intensive Care.

[13] Textor, Stephen C, and Lilach Lerman. Renovascular hypertension and ischemic nephropathy. Am J Hypertens. 2010v;23(11):1159–69. doi: 10.1038/ajh.2010.174. 10.1038/ajh.2010.174 PMC3078640 20864945

[14] Lobeck IN, Alhajjat AM, Dupree P, Racadio JM, Mitsnefes MM, Karns R (2018). The management of pediatric renovascular hypertension: a single center experience and review of the literature. J Pediatr Surg.

[15] Agrawal H, Moodie D, Qureshi AM, Acosta AA, Hernandez JA, Braun MC (2018). Interventions in children with renovascular hypertension: A 27-year retrospective single-center experience. Congenit Heart Dis.

[16] Wu HP, Yang WC, Wu YK, Zhao LL, Chen CY, Fu YC (2012). Clinical significance of blood pressure ratios in hypertensive crisis in children. Arch Dis Child.

[17] Shroff R, Roebuck DJ, Gordon I, Davies R, Stephens S, Marks S (2006). Angioplasty for renovascular hypertension in children: 20-year experience. Pediatrics.

[18] Deal JE, Barratt TM, Dillon MJ (1992). Management of hypertensive emergencies. Arch Dis Child.

[19] Tullus K, Roebuck DJ, McLaren CA, Marks SD (2010). Imaging in the evaluation of renovascular disease. Pediatr Nephrol.

[20] Sandmann W, Dueppers P, Pourhassan S, Voiculescu A, Klee D, Balzer KM (2014). Early and long-term results after reconstructive surgery in 42 children and two young adults with renovascular hypertension due to fibromuscular dysplasia and middle aortic syndrome. Eur J Vasc Endovasc Surg.

[21] McCulloch M, Andronikou S, Goddard E, Sinclair P, Lawrenson J, Mandelstam S (2003). Angiographic features of 26 children with Takayasu's arteritis. Pediatr Radiol.

[22] Bayrak AH, Numan F, Cantaşdemir M, Baş A (2008). Percutaneous balloon angioplasty of renovascular hypertension in pediatric cases. Acta Chir Belg.

[23] Zhu G, He F, Gu Y, Yu H, Chen B, Hu Z (2014). Angioplasty for pediatric renovascular hypertension: a 13-year experience. Diagn Interv Radiol.

[24] Peco-Antić A, Stajić N, Krstić Z, Bogdanović R, Miloševski-Lomić G, Đukić M (2016). Associated extrarenal vascular diseases may complicate the treatment and outcome of renovascular hypertension. Acta paediatrica.

[25] De Bruyne B, Manoharan G, Pijls NH, Verhamme K, Madaric J, Bartunek J (2006). Assessment of renal artery stenosis severity by pressure gradient measurements. J Am Coll Cardiol.

